# Supplementary material for: An archetype and scaling of developmental tissue dynamics across species
Source: Nat Commun. 2023 Dec 11;14:8199. doi: 10.1038/s41467-023-43902-y (PMC10713982; doi:10.1038/s41467-023-43902-y)
Supplement: Supplementary file 1 — Supplementary Information [file 41467_2023_43902_MOESM1_ESM.pdf]

Supplementary Information for

An archetype and scaling of developmental tissue dynamics across species

Yoshihiro Morishita*, Sang-Woo Lee, Takayuki Suzuki, Hitoshi Yokoyama, Yasuhiro Kamei, Koji Tamura, Aiko Kawasumi-Kita

*Corresponding author. Email: yoshihiro.morishita@riken.jp

This PDF file includes:

Figs. S1 to S7
Legends for Supplementary figures

Figure S1

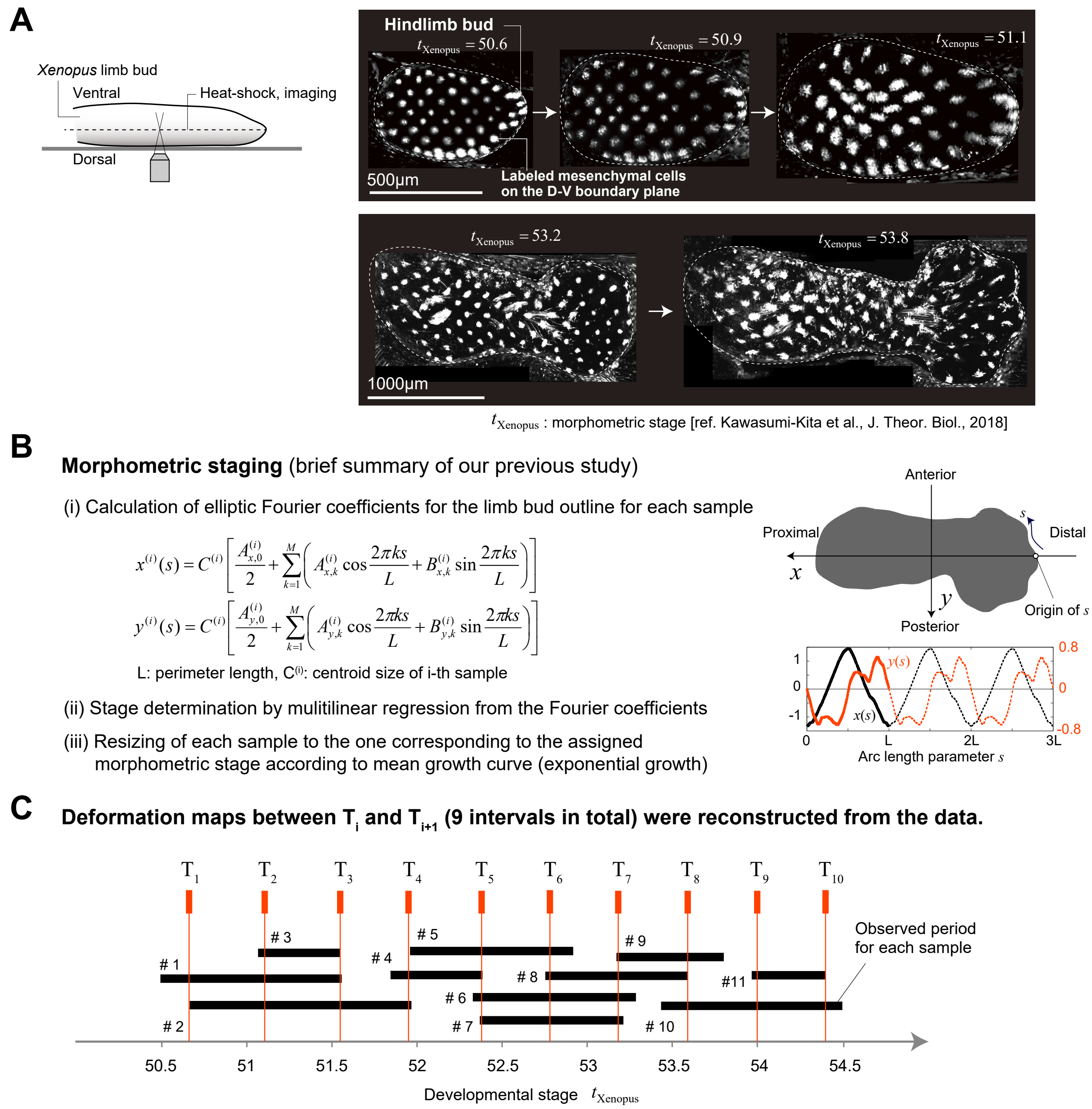

Figure S1: Data and preprocessing for reconstruction of the tissue deformation map during *Xenopus* hindlimb development

(**A**) Two examples used for the lineage tracing of mesenchymal cells labeled by heat shock induction of EGFP (bottom) on the frontal plane around the mid dorso-ventral level (top). The labeled spots are sufficiently spaced apart to enable each lineage to be followed.

(**B**) A brief summary of the morphometric staging proposed in our previous study (21). The right images were adapted from Kawasumi et al 2018 (ref 21) under a CC-BY licence. The figure has been slightly modified.

(**C**) Lineage tracing data from 11 samples were used to reconstruct the tissue deformation map from the initial limb bud phase through the stage at which cartilage patterning is complete. The developmental stages for each measurement were different among the samples, although some overlapped with each other. We first reconstructed the deformation maps for nine time intervals, then integrated them to obtain the full map spanning the duration of our time of interest.

Figure S2

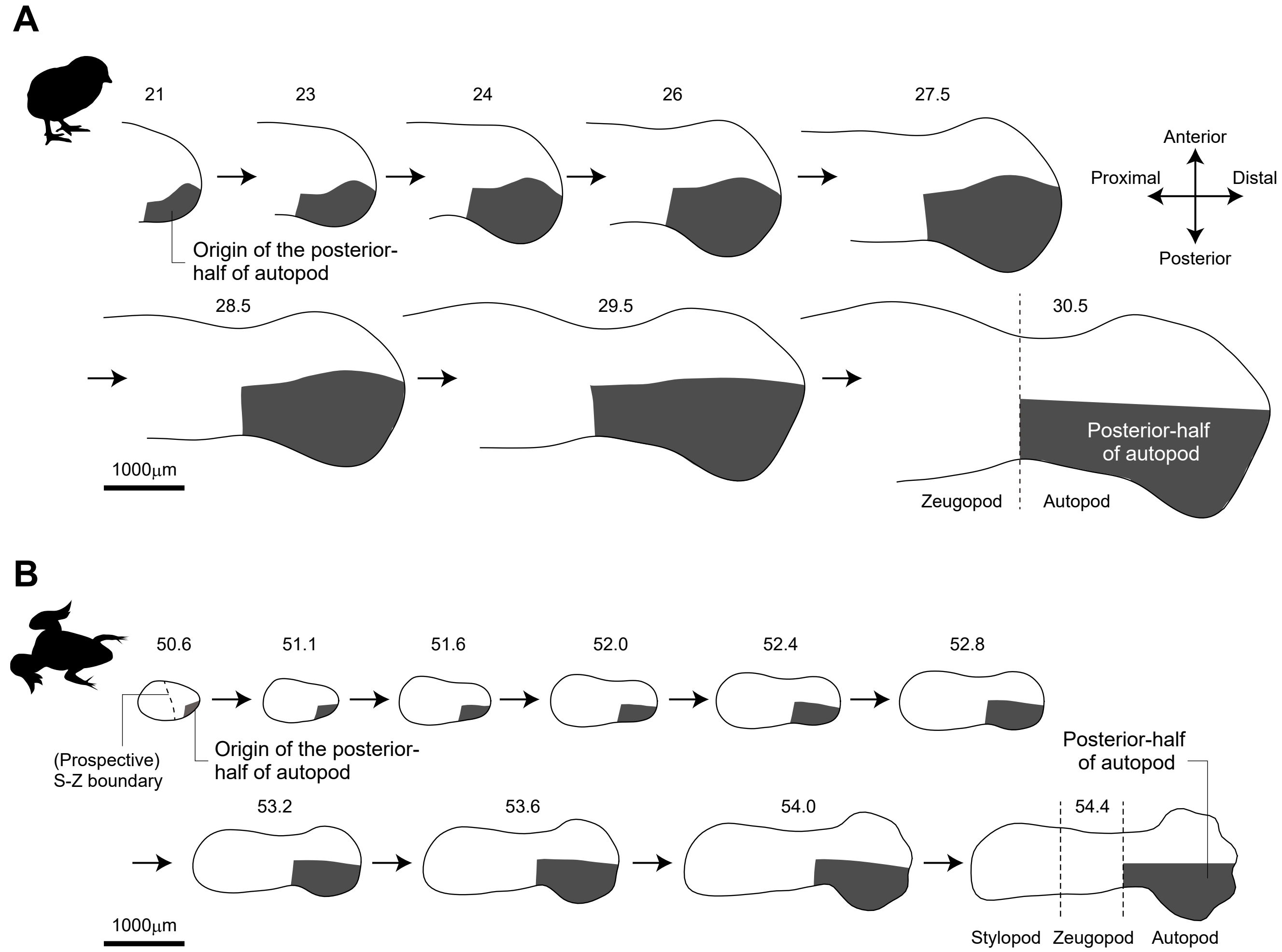

Figure S2: Origins and lineages of the posterior-half of the autopod

(**A**, **B**) The origins and lineage of the posterior-half of the autopod were calculated by inverse-mapping of the tissue deformation maps for chick (**A**) or *Xenopus* (**B**) hindlimb development.

Figure S3

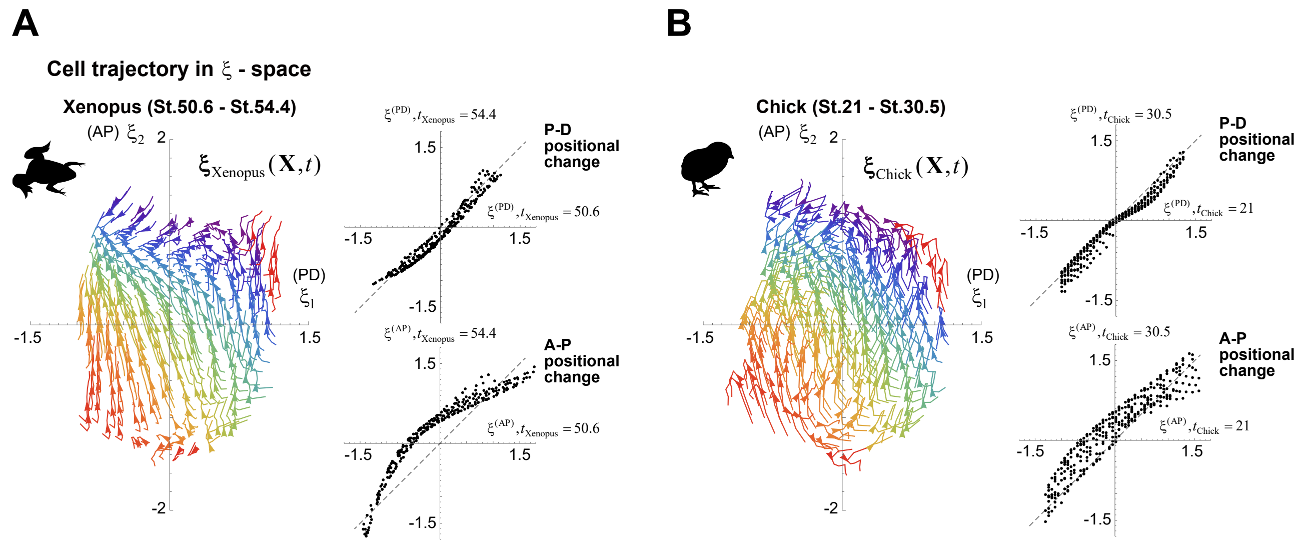

Figure S3: Cell trajectories in ξ-space during *Xenopus* and chick limb development (for the full time periods) (**A, B**) (left) Cell trajectories in the ξ-space for *Xenopus* (**A**) and chick (**B**) limb morphogenesis and (right) the positional changes in the proximo-distal (P-D) or antero-posterior (A-P) direction in the ξ-space between an early stage and a stage at which digit patterning and cartilage differentiation is complete ($t_{Xenopus}$ =54.4 for *Xenopus* and t_{Chick} =30.5 for chick). The differences in cell trajectories and orientation of the velocity field between the two species become clear around the stages of chondrogenic differentiation after initial digit patterning with spatial heterogeneity in cell density (i.e., after $t_{Xenopus}$ =53.5 and t_{Chick} =27-27.5). For both species, trajectories of the same color indicate trajectories from the same initial position. Note that the dynamics for the prospective autopod and zeugopod are compared.

Figure S4

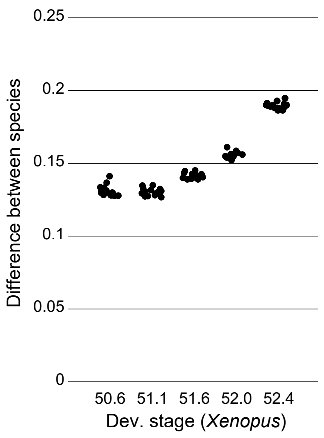

Figure S4: Dependence of the agreement in cell trajectories in the ξ-space between species on the selection of initial stages

The results for the case in which the initial time point for chick was fixed at t_{Chick} =21 and the five initial time points for *Xenopus* ($t_{Xenopus}$ =50.6, 51.1, 51.6, 52.0, 52.4) were tested are shown. (Median, SD)=(0.130, 0.0037), (0.130, 0.0025), (0.141, 0.0019), (0.155,0.0022), (0.190,0.9922) for $t_{Xenopus}$ =50.6, 51.1, 51.6, 52.0, 52.4, respectively.

Figure S5

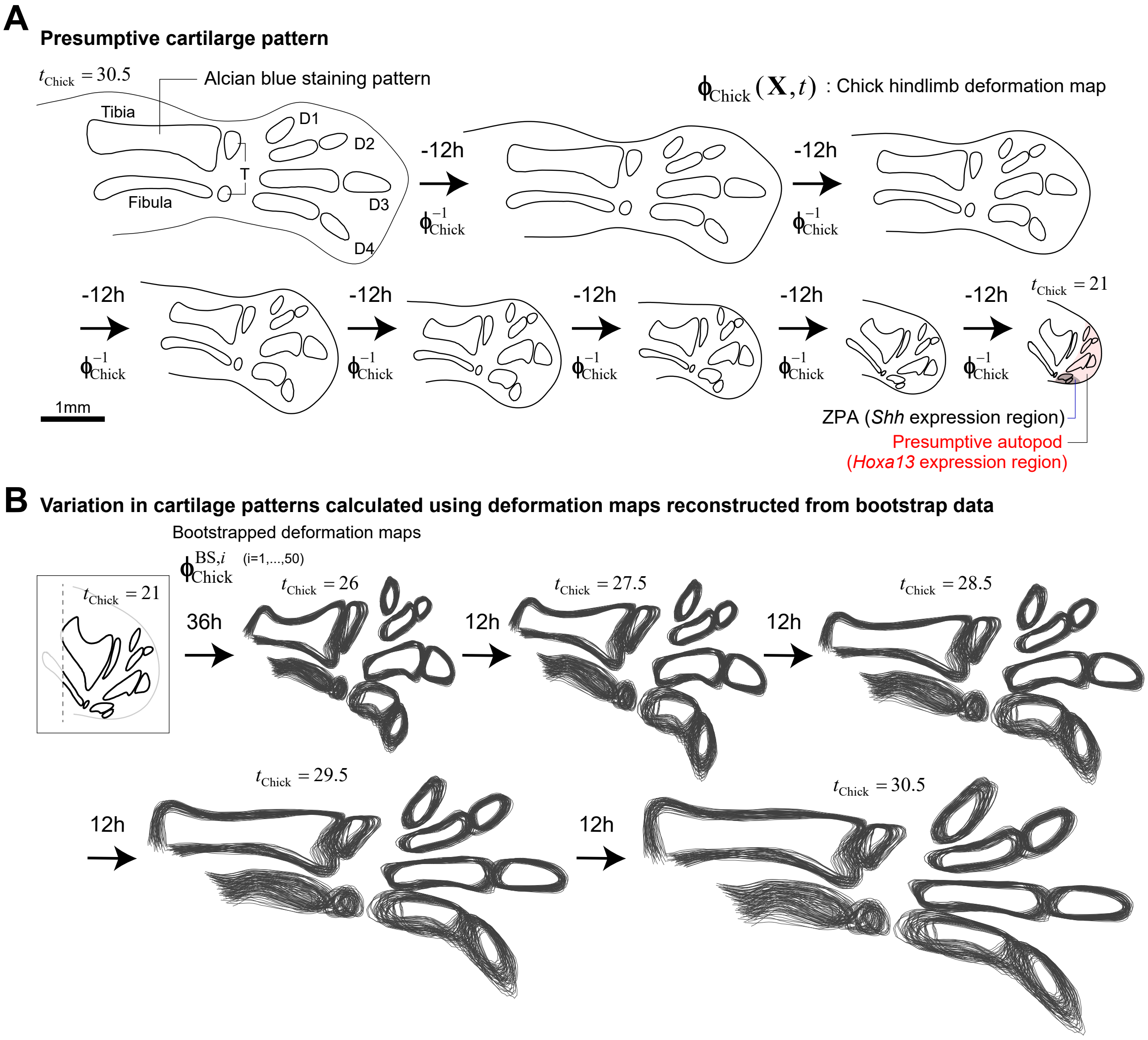

Figure S5: Calculation of the presumptive skeletal patterns and bootstrapped maps.

(**A**) Inverse images of the cartilage differentiation pattern obtained by Alcian blue staining at t_{Chick} =30.5 were calculated using the reconstructed deformation map $\phi_{\text{Chick}}(\boldsymbol{X}, t)$. The prospective autopod region at the initial limb bud (t_{Chick} =21) matched well with the region of hoxa13 expression, a typical marker gene of the autopod. Prospective digit 4 was included in the Shh expression region at the zone of polarizing activity (ZPA). D1-D4: digits (phalanges and metatarsals); T: tarsals.

(**B**) Bootstrapped maps were generated as a way to assess intraspecies variability (Fig. 5**A**, Materials and Methods). In (**B**), to visually understand the variability, (prospective) skeletal patterns calculated using 50 different bootstrapped maps are shown.

Figure S6

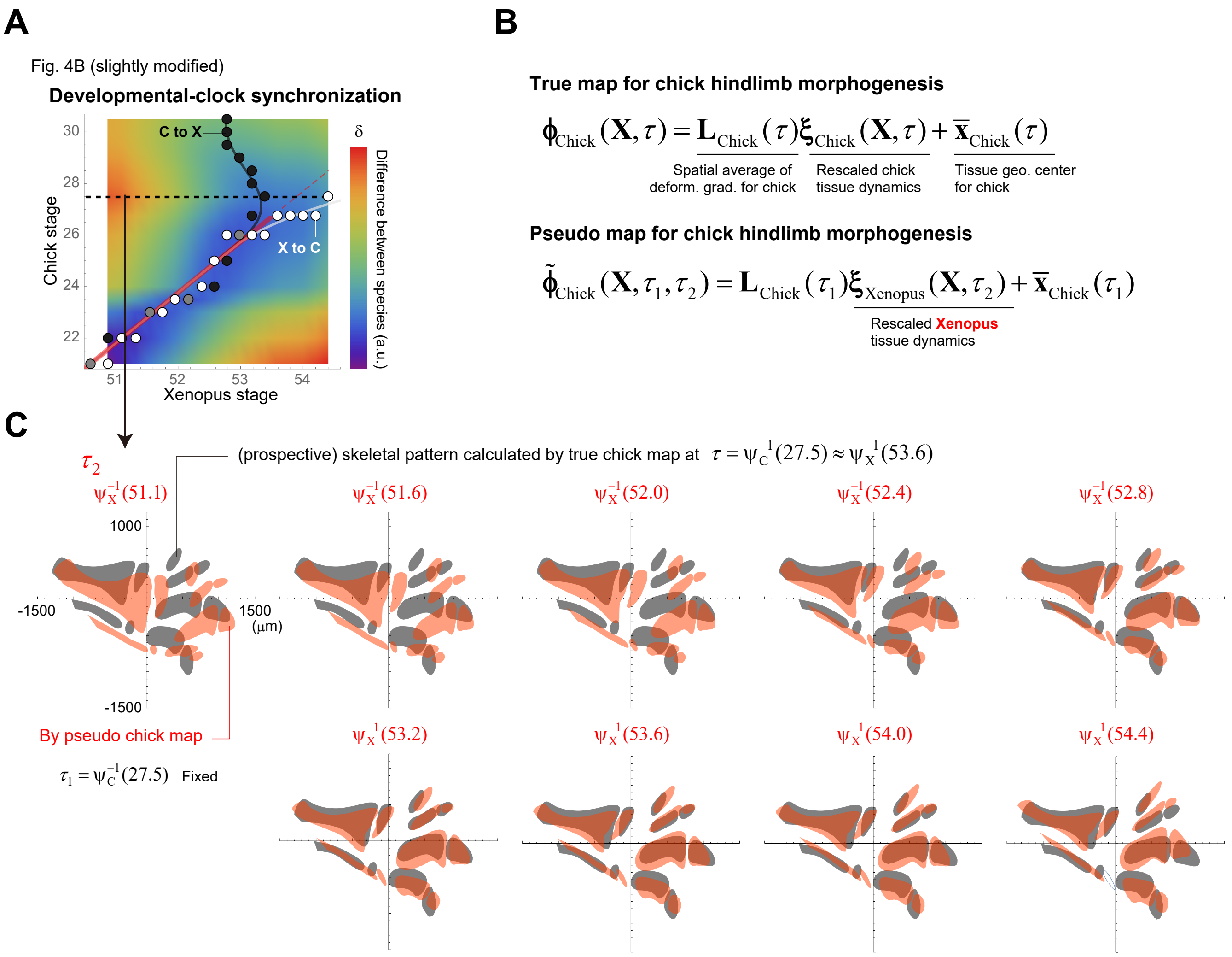

Figure S6: Comparison of true and pseudo chick limb deformation maps

(**A**) A slightly modified version of Fig. 4(**B**).

(**B**) Definitions for the true and pseudo maps.

(**C**) Comparison of (prospective) skeletal patterns calculated by the true (gray) and pseudo (red) maps. The pattern calculated by the true map is for the stage 27.5, i.e., $\tau = \psi_C^{-1}(27.5)$. For the pseudo map, the images at nine timepoints (τ_2) were calculated as shown along the black dotted line in (**A**).

Figure S7

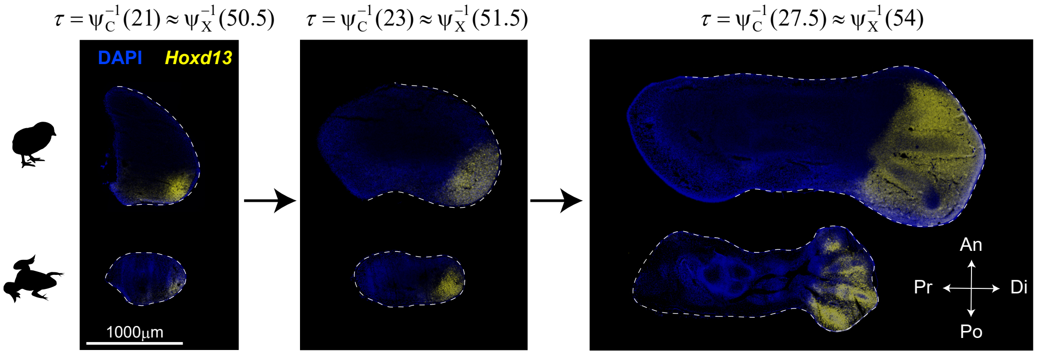

Figure S7: Spatiotemporal expression patterns of the *hoxd13* gene

(**A**) The expression patterns were obtained by RNAscope assays at three different timepoints on the common clock (represented under fixed ordinary Cartesian coordinates). These data were used to calculate the ξ-representation of their expression patterns in Fig. 6(**B**).
