# Supplementary material for: An archetype and scaling of developmental tissue dynamics across species
Source: Nat Commun. 2023 Dec 11;14:8199. doi: 10.1038/s41467-023-43902-y (PMC10713982; doi:10.1038/s41467-023-43902-y)
Supplement: Supplementary file 3 — Description of Additional Supplementary Files [file 41467_2023_43902_MOESM3_ESM.pdf]

Description of Additional Supplementary Files

Supplementary Software 1: This code is for calculating cell trajectories in the ξ coordinate system from the positional vectors of each cell within the tissue at each time point under the ordinary Cartesian coordinate system and the deformation gradient tensor at that location, which represents local tissue deformation.
