# Supplementary material for: An archetype and scaling of developmental tissue dynamics across species
Source: Nat Commun. 2023 Dec 11;14:8199. doi: 10.1038/s41467-023-43902-y (PMC10713982; doi:10.1038/s41467-023-43902-y)
Supplement: Supplementary file 5 — Reporting Summary [file 41467_2023_43902_MOESM5_ESM.pdf]

nature portfolio

Corresponding author(s): Yoshihiro Morishita

Last updated by author(s): Nov 6, 2023

Reporting Summary

Nature Portfolio wishes to improve the reproducibility of the work that we publish. This form provides structure for consistency and transparency in reporting. For further information on Nature Portfolio policies, see our Editorial Policies and the Editorial Policy Checklist.

Statistics

For all statistical analyses, confirm that the following items are present in the figure legend, table legend, main text, or Methods section.

n/a	Confirmed	
☐	☒	The exact sample size (*n*) for each experimental group/condition, given as a discrete number and unit of measurement
☐	☒	A statement on whether measurements were taken from distinct samples or whether the same sample was measured repeatedly
☒	☐	The statistical test(s) used AND whether they are one- or two-sided<br>*Only common tests should be described solely by name; describe more complex techniques in the Methods section.*
☒	☐	A description of all covariates tested
☒	☐	A description of any assumptions or corrections, such as tests of normality and adjustment for multiple comparisons
☐	☒	A full description of the statistical parameters including central tendency (e.g. means) or other basic estimates (e.g. regression coefficient) AND variation (e.g. standard deviation) or associated estimates of uncertainty (e.g. confidence intervals)
☒	☐	For null hypothesis testing, the test statistic (e.g. *F*, *t*, *r*) with confidence intervals, effect sizes, degrees of freedom and *P* value noted<br>*Give P values as exact values whenever suitable.*
☐	☒	For Bayesian analysis, information on the choice of priors and Markov chain Monte Carlo settings
☒	☐	For hierarchical and complex designs, identification of the appropriate level for tests and full reporting of outcomes
☒	☐	Estimates of effect sizes (e.g. Cohen's *d*, Pearson's *r*), indicating how they were calculated

Our web collection on statistics for biologists contains articles on many of the points above.

Software and code

Policy information about availability of computer code

Data collection	No software was used for data collection.
Data analysis	Fiji ImageJ, Mathematica<br>The code used for calculating the xi coordinates as proposed in this paper is available as a Mathematica file in the Supplementary information (import data is also included).

For manuscripts utilizing custom algorithms or software that are central to the research but not yet described in published literature, software must be made available to editors and reviewers. We strongly encourage code deposition in a community repository (e.g. GitHub). See the Nature Portfolio guidelines for submitting code & software for further information.

Data

Policy information about availability of data

All manuscripts must include a data availability statement. This statement should provide the following information, where applicable:

- Accession codes, unique identifiers, or web links for publicly available datasets
- A description of any restrictions on data availability
- For clinical datasets or third party data, please ensure that the statement adheres to our policy

All source data are provided with this paper in the Supplementary information.

Research involving human participants, their data, or biological material

Policy information about studies with human participants or human data. See also policy information about sex, gender (identity/presentation), and sexual orientation and race, ethnicity and racism.

Reporting on sex and gender	N/A
Reporting on race, ethnicity, or other socially relevant groupings	N/A
Population characteristics	N/A
Recruitment	N/A
Ethics oversight	N/A

Note that full information on the approval of the study protocol must also be provided in the manuscript.

Field-specific reporting

Please select the one below that is the best fit for your research. If you are not sure, read the appropriate sections before making your selection.

[x] Life sciences   [ ] Behavioural & social sciences   [ ] Ecological, evolutionary & environmental sciences

For a reference copy of the document with all sections, see nature.com/documents/nr-reporting-summary-flat.pdf

Life sciences study design

All studies must disclose on these points even when the disclosure is negative.

Sample size	In the reconstruction of tissue deformation maps, 11 Xenopus embryos and the following numbers of fluorescently labeled cells were used. St.50.6-51.1: N=113; St.51.1-51.6: N=185; St.51.6-52.0: N=67; St.52.0-52.4: N=79; St.52.4-52.8: N=226; St.52.8-53.2: N=333; St.53.2-53.6: N=256; St.53.6-54.0: N=129; St.54.0-54.4: N=270
Data exclusions	No data were excluded from the analyses.
Replication	Regarding RNAscope assay and H&E/Alcian-Blue staining, each experiment was independently repeated three times with similar results. We also verified the similarity of cell trajectories across embryos during overlapping measurement periods.
Randomization	Embryos were selected at random for imaging and gene expression analysis.
Blinding	N/A

Reporting for specific materials, systems and methods

We require information from authors about some types of materials, experimental systems and methods used in many studies. Here, indicate whether each material, system or method listed is relevant to your study. If you are not sure if a list item applies to your research, read the appropriate section before selecting a response.

Materials & experimental systems

n/a	Involved in the study	
[x]	[ ]	Antibodies
[x]	[ ]	Eukaryotic cell lines
[x]	[ ]	Palaeontology and archaeology
[ ]	[x]	Animals and other organisms
[x]	[ ]	Clinical data
[x]	[ ]	Dual use research of concern
[x]	[ ]	Plants

Methods

n/a	Involved in the study	
[x]	[ ]	ChIP-seq
[x]	[ ]	Flow cytometry
[x]	[ ]	MRI-based neuroimaging

Animals and other research organisms

Policy information about studies involving animals; ARRIVE guidelines recommended for reporting animal research, and Sex and Gender in Research

Laboratory animals	Xenopus laevis: (1) wild type (purchased from a domestic animal vendor), (2) Xla.Tg (Xla.hsp70:eGFP), gifted from Dr. Yumi Izutsu. Chick: wild type (purchased from a domestic animal vendor) Animal ages: St. 50-55 (Xenopus laevis tadpoles), St. 20-30.5 (chick embryos)
Wild animals	The study did not involve wild animals.
Reporting on sex	All experiments were conducted during larval stages long before sexual maturity.
Field-collected samples	The study did not involve samples collected from the field.
Ethics oversight	Experiments on Xenopus limb development were conducted at the National Institute of Basic Biology (NIBB), Tohoku University, and RIKEN Center for Biosystems Dynamics Research (RIKEN BDR). All procedures and protocols were approved by the Institutional Animal Care and Use Committee of NIBB. RIKEN and Tohoku University (and also Japanese domestic law, Act on Welfare and Management of Animals) exempt studies involving amphibians from requiring IRB approval, however, all experiments at RIKEN BDR and Tohoku University were performed in accordance with NIBB's ethics guidelines. Regarding chick embryos (wild type), we purchased fertilized chicken eggs from Shiroyama Farm and Inoue egg farm. The experiments comply with all ethical regulations because no licensing was required to study chick embryos at the stages used (HH20-HH30.5).

Note that full information on the approval of the study protocol must also be provided in the manuscript.
